# Domino reactions of chromones with activated carbonyl compounds

**DOI:** 10.3762/bjoc.20.108

**Published:** 2024-05-29

**Authors:** Peter Langer

**Affiliations:** 1 Institut für Chemie, Universität Rostock, Albert-Einstein-Str. 3a, 18059 Rostock, Germany; 2 Leibniz-Institut für Katalyse e. V. an der Universität Rostock, Albert-Einstein-Str. 29a, 18059 Rostock, Germany

**Keywords:** cyclizations, 1,3-dicarbonyl compounds, domino reactions, heterocycles, regioselectivity, silyl enol ethers

## Abstract

Domino reactions of chromones with activated carbonyl compounds, such as dimethyl acetone-1,3-dicarboxylate and 1,3-diphenylacetone, and with 1,3-bis(silyloxy)-1,3-butadienes, electroneutral equivalents of 1,3-dicarbonyl dianions, allow for a convenient synthesis of a great variety of products. The regioselectivity and course of the reaction depends of the substituent located at carbon C3 of the chromone moiety and also on the type of nucleophile employed.

## Introduction

**Scheme 1:**
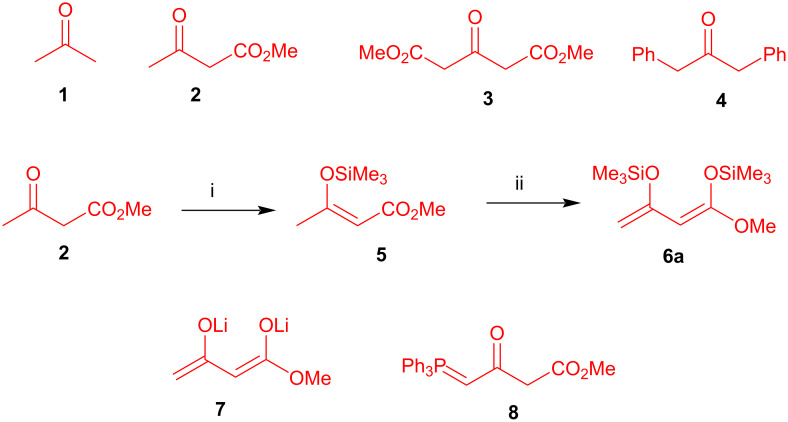
Structures of carbonyl compounds **1**, **2, 3,** and **4**, dianion **7**, phosphorane **8** and synthesis of 1,3-bis(silyloxy)-1,3-butadiene **6a** via **5**. Conditions: i, Me_3_SiCl, toluene, NEt_3_, 24 h; ii, 1) LDA, THF, −78 °C, 1 h; 2) Me_3_SiCl, −78 to 20 °C, 12 h.

Domino reactions (also called cascade or tandem reactions) allow for the synthesis of complex products with formation of several bonds in only one step [1–5]. In contrast to non-activated acetone (**1a**), methylene-active compounds, such as 1,3-dicarbonyl compounds, are of considerable relevance as building blocks in domino reactions (Scheme 1). For example, methyl acetoacetate (**2a**) possesses one CH-acidic methylene carbon which can be deprotonated in equilibrium by weak organic bases, such as triethylamine, piperidine or DBU, under thermodynamic conditions. Dimethyl acetone-1,3-dicarboxylate (**3a**) contains even two reactive methylene groups which can undergo cyclization reactions under mild conditions. In case of 1,3-diphenylacetone (**4a**) some activation of the methylene group is observed as well, because of benzylic stabilization.

Dianions of 1,3-dicarbonyl compounds follow a different regioselectivity as compared to simple monoanions [6–12]. For example, **2** can be transformed to its dianion **7** by action of two equivalents of LDA or by sequential addition of sodium hydride and *n*-butyllithium (Scheme 1). In contrast to **2**, dianion **7** reacts with electrophiles at its terminal carbon atom. 1,3-Bis(silyloxy)-1,3-butadienes, containing two silyl enol ether moieties, can be regarded as masked dianions or electroneutral equivalents of 1,3-dicarbonyl dianions and react with the same regioselectivity [13–22]. Both free and masked dianions were studied extensively in my group for many years. 1,3-Bis(silyloxy)-1,3-butadienes are highly moisture sensitive compounds which can be prepared from the corresponding 1,3-dicarbonyl dianions in two steps. For example, diene **6a** is available by silylation of **2** to give silyl enol ether **5** which is subsequently deprotonated by LDA and treated with trimethylchlorosilane. A direct preparation of **6a** by reaction of dianion **7** with trimethylchlorosilane is not possible, because the silylation occurs at the terminal carbon atom of the dianion. (2,4-Dioxobutylidene)phosphorane **8** also represents a synthetic equivalent of dianion **7** [23]. However, reactions of such phosphoranes were less studied in my group.

**Scheme 2:**
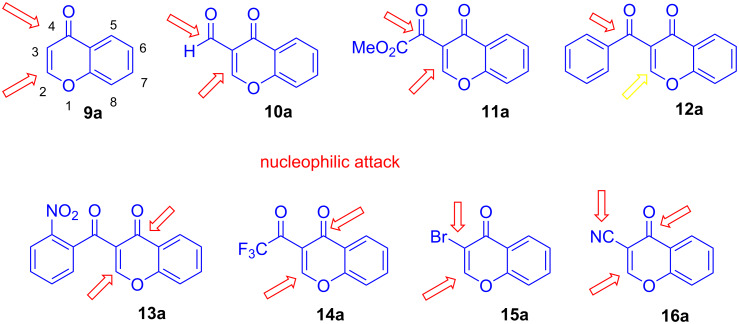
Structures of chromones with different substituents located at carbon C-3 and atom numbering scheme exemplified for **9a**.

Chromones are oxygen heterocycles which are of pharmacological interest and represent versatile synthetic building blocks which can undergo various types of domino reactions [24–28]. In 2001, Nehad N. R. Saleh in my group developed a new synthesis of benzocoumarines by reaction of 1,3-bis(silyloxy)-1,3-butadiene **6a** with parent 2,3-unsubstituted chromone (**9a**). This marked the starting point of chromone chemistry in my group. In the following years, we studied reactions of various types of chromones with 1,3-bis(silyloxy)-1,3-butadienes [29]. Starting in 2010, we studied also cyclization reactions of chromones with heterocyclic enamines [30–31]. The present review aims to provide a personalized account of our work related to domino reactions of various types of chromones with carbonyl compounds, such as **3** and **4**, and with 1,3-bis(silyloxy)-1,3-butadienes, such as **6a**. These studies include various types of chromones, including 2,3-unsubstituted chromone (**9a**), 3-formylchromone (**10a**), 3-acetylchromone, 3-(methoxalyl)chromone (**11a**), 3-benzoylchromone (**12a**), 3-(2-nitrobenzoyl)chromone (**13a**), 3-trifluoroacetylchromone (**14a**), 3-perfluoroalkanoylchromones, 3-bromochromone (**15a**), 3-chloro- and 3-iodochromone, and 3-cyanochromone (**16a**) (Scheme 2). Depending on the type of chromone and nucleophile, the reactions follow a different regioselectivity.

## Review

### 2,3-Unsubstituted chromones

#### Dimethyl acetone-1,3-dicarboxylate

**Scheme 3:**
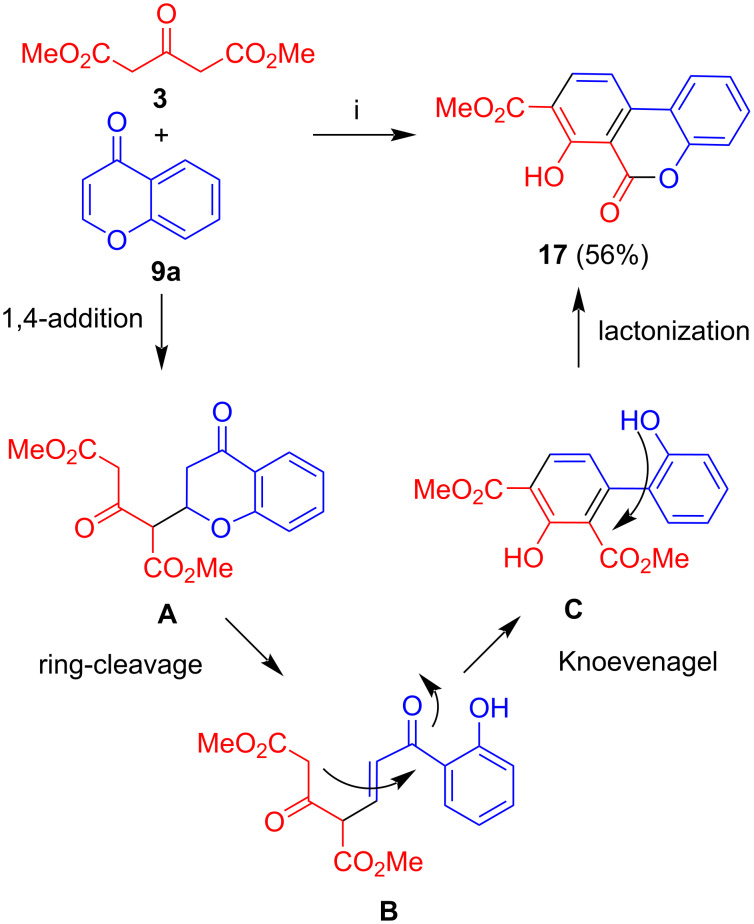
Synthesis of **17**. Conditions: i, DBU (1.3 equiv), THF, 20 °C, 12 h.

The base-mediated reaction of dimethyl acetone-1,3-dicarboxylate (**3**) with 2,3-unsubstituted parent chromone (**9a**) afforded benzocoumarine **17** in 56% yield (Scheme 3) [32]. The formation of the product can be explained by 1,4-addition of **3** to the chromone to give intermediate **A** and ring cleavage to give intermediate **B**. Subsequent Knoevenagel reaction by attack of the methylene carbon to the carbonyl group gave intermediate **C** which underwent lactonization to give the final product.

#### 1,3-Bis(silyloxy)-1,3-butadienes

**Scheme 4:**
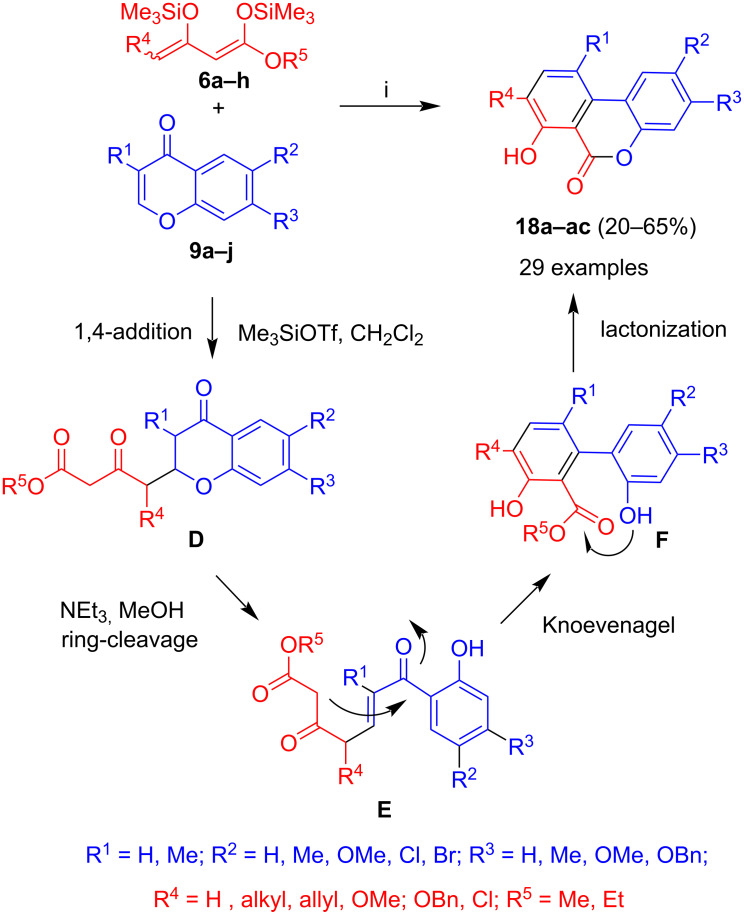
Synthesis of **18a–ac**. Conditions: i, 1) **9a–j**, Me_3_SiOTf (1.3 equiv), 20 °C, 1 h; 2) **6a–h** (1.3 equiv), CH_2_Cl_2_, 0 → 20 °C, 12 h; 3) HCl (10%); 4) NEt_3_ (2.0 equiv), EtOH, 20 °C, 12 h.

The reaction of chromones **9a–j** with 1,3-bis(silyloxy)-1,3-butadienes **6a–h**, carried out in the presence of Me_3_SiOTf, and subsequent treatment with triethylamine in methanol afforded benzocoumarines **18a–ac** (Scheme 4) [33–34]. The formation of the product, which was obtained in a two-step one-pot reaction, can be explained by a mechanism similar to the one discussed for the formation of **17**. 1,4-Addition gave intermediate **D** which was isolated, but used in crude form for the next step. Treatment of the latter with triethylamine in methanol resulted in ring-cleavage to give intermediate **E**. Subsequent base-mediated Knoevenagel reaction with extrusion of water gave intermediate **F** which underwent lactonization and resulted in the formation of the final product. The reaction proved to be rather general and various dienes and chromones were successfully employed to give the corresponding benzozocoumarines in good yields. The yields were in most of the cases in the range of 42 to 65% (over two steps) and did not depend much on the substituents attached to the chromone and at carbon C-4 of the diene. However, in case of a benzyl and a benzyloxy group located at carbon C-4 of the diene, the overall yields were rather low (20%). This might be explained by steric effects. One of the best yields was obtained for the reaction of diene **6a** with unsubstituted chromone **9a** and for the diene containing a methoxy group (R^4^ = OMe). The products proved to be interesting, due to their blue fluorescence.

### 3-Formylchromones

#### Dimethyl acetone-1,3-dicarboxylate

**Scheme 5:**
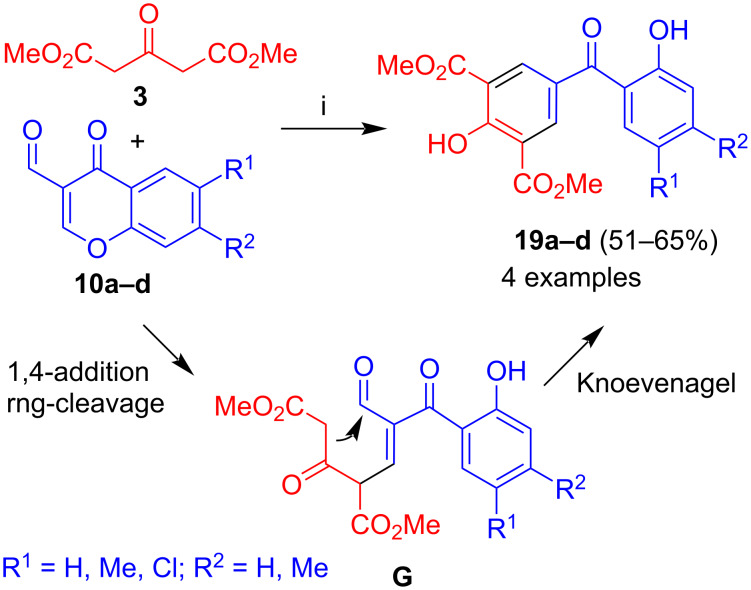
Synthesis of **19a–d**. Conditions: i, DBU (1.3 equiv), THF, 20 °C, 12 h.

The reaction of **3** with 3-formylchromones **10a–d** afforded benzophenones **19a–d** in moderate to good yields (Scheme 5) [32]. The formation of the products can be explained by Michael reaction (1,4-addition) of **3** to the chromone and ring cleavage to give intermediate **G**. Subsequent Knoevenagel reaction by attack of the methylene carbon to the aldehyde resulted in the formation of the final products. The regioselective cyclization can be explained by the higher electrophilicity of the aldehyde as compared to the ketone. The yields were in general quite good (51–65%). Relatively low yields (51–52%) were obtained for chromones containing methyl substituents, presumably due to the lower electrophilicity of the chromone based on the electron-donating effect of the methyl groups. Good yields were obtained for parent 3-formylchromone (**10a**) and for chlorine-substituted chromones, because of the higher electrophilicity of the chromone based on the electron-withdrawing effect of the methyl groups.

#### 1,3-Bis(silyloxy)-1,3-butadienes

**Scheme 6:**
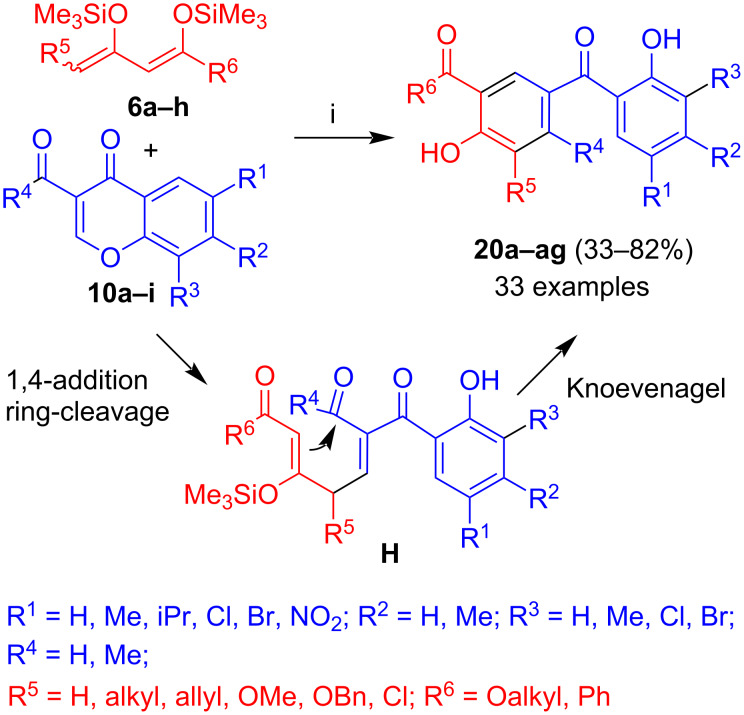
Synthesis of **20a–ag**. Conditions: i, 1) **10a–i**, Me_3_SiOTf (0.3 equiv), 20 °C, 10 min; 2) **6a–h** (1.3 equiv), CH_2_Cl_2_, 0 → 20 °C, 12 h; 3) HCl (10%).

The reaction of 3-formyl- and 3-acetylchromones **10a–i** with 1,3-bis(silyloxy)-1,3-butadienes **6a–h**, catalysed by Me_3_SiOTf, afforded hydroxylated benzophenones **20a–ag** (Scheme 6) [35–36]. The products are formed by a mechanism related to the one discussed for the formation of products **19a–d**. The same type of product is formed in the reaction of 3-formylchromones with (2,4-dioxobutylidene)phosphoranes [37]. The yields were in a range of 33 to 82%. No clear trend was observed for the yields depending on the structure of the diene and the chromone. Similar to unsubstituted diene **6a**, dienes containing a substituent R^5^ located at carbon C-4 gave good yields, except for methoxy and benzyloxy which did not work at all. Dienes derived from 1,3-diketones (R^6^ = Me, Ph) gave similar yields to those obtained from dienes derived form β-ketoesters, although yields were slightly lower in case of R^6^ = Me. The best yield was obtained for the cyclization of unsubstituted diene **6a** with a chlorine-substituted chromone, presumably because of the electron-withdrawing character of the chlorine atoms and, thus, higher electrophilicity of the chromone.

### 3-(Methoxalyl)chromones

#### Dimethyl acetone-1,3-dicarboxylate

**Scheme 7:**
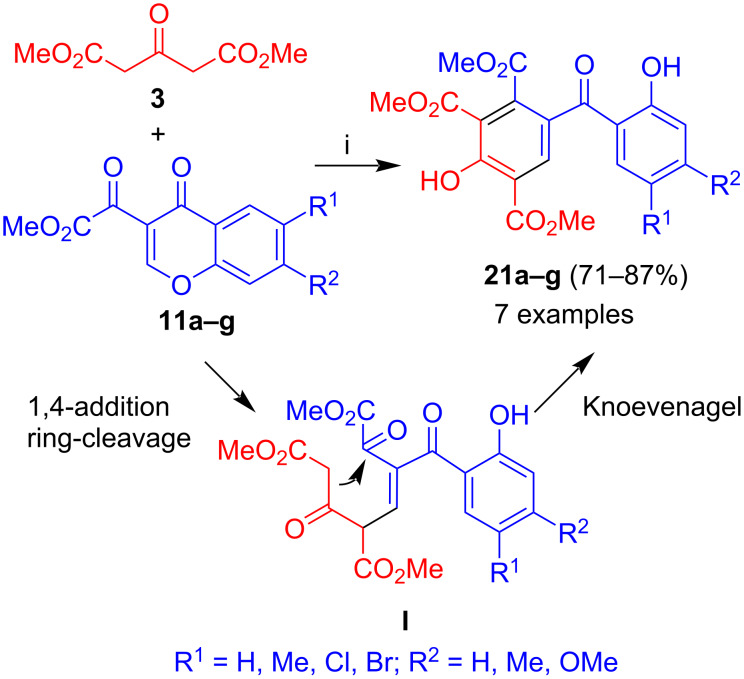
Synthesis of **21a–g**. Conditions: i, DBU (1.3 equiv), dioxane, 20 °C, 12 h.

The reaction of dimethyl acetone-1,3-dicarboxylate (**3**) with 3-(methoxalyl)chromones **11a–g**, a new type of chromone developed in our group, afforded benzophenones **21a–g** in moderate to very good yields (Scheme 7) [38]. The formation of the products can be explained by Michael reaction (1,4-addition) of **3** to the chromone and ring cleavage to give intermediate **I**. Subsequent Knoevenagel reaction by attack of the methylene carbon to the carbonyl group adjacent to the ester group resulted in the formation of the final products. The regioselective cyclization can be explained by the higher electrophilicity of the carbonyl group adjacent to the ester, due to the electron-withdrawing effect of the latter. The yields were generally good to very good and no trend was observed depending on the structure of the chromones.

#### 1,3-Diphenylacetone

**Scheme 8:**
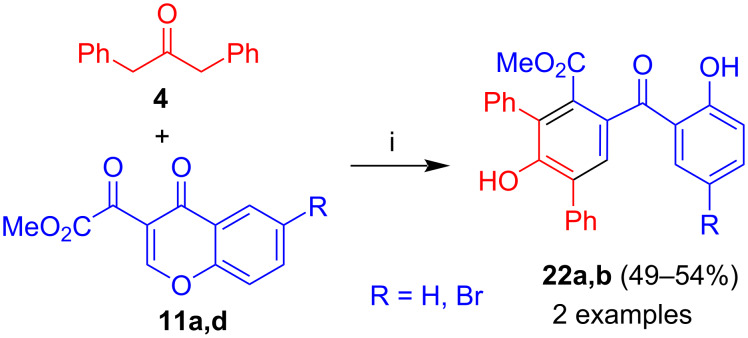
Synthesis of **22a,b**. Conditions: i, DBU (1.3 equiv), dioxane, 20 °C, 12 h.

Similarly, reaction of 1,3-diphenylacetone (**4**) with **11a**,**d** afforded benzophenones **22a**,**b** in moderate yields (Scheme 8) [38]. Again, no trend was observed depending on the structure of the chromones.

#### 1,3-Bis(silyloxy)-1,3-butadienes

**Scheme 9:**
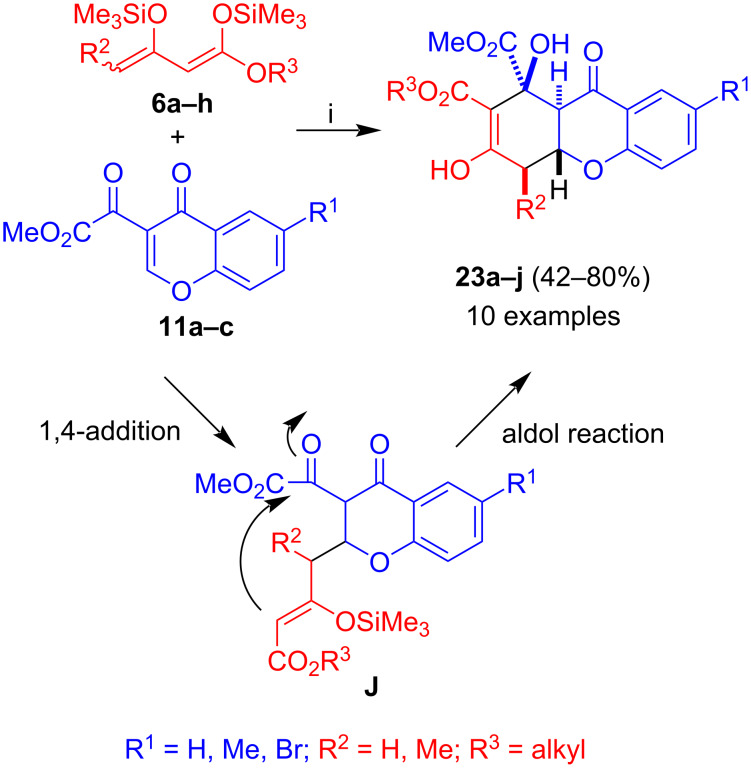
Synthesis of **23a–j**. Conditions: i, 1) **11a–c**, Me_3_SiOTf (0.3 equiv), 20 °C, 1 h; 2) **6a–h** (1.3 equiv), CH_2_Cl_2_, 0 → 20 °C, 12 h; 3) HCl (10%).

The reaction of **11a–c** with 1,3-bis(silyloxy)-1,3-butadienes **6a–h**, catalyzed by Me_3_SiOTf, afforded products **23a–j** with excellent diastereoselectivity (Scheme 9) [39]. The formation of the products can be explained by 1,4-addition of the terminal carbon of the diene to the chromone to give intermediate **J** and subsequent attack of the central carbon atom of the 1,3-dicarbonyl unit (aldol reaction). No aromatization and extrusion of the hydroxy group was observed under these conditions, because the electron-withdrawing character of the additional ester group which destabilizes formation of a cation at the neighboring position. The yields were in most cases moderate (42–54%). Two products were obtained in higher yields (71 and 80%). However, no clear trend was observed for the yields depending on the substitution pattern. Instead, the individual purity and quality of the dienes played an important role.

**Scheme 10:**
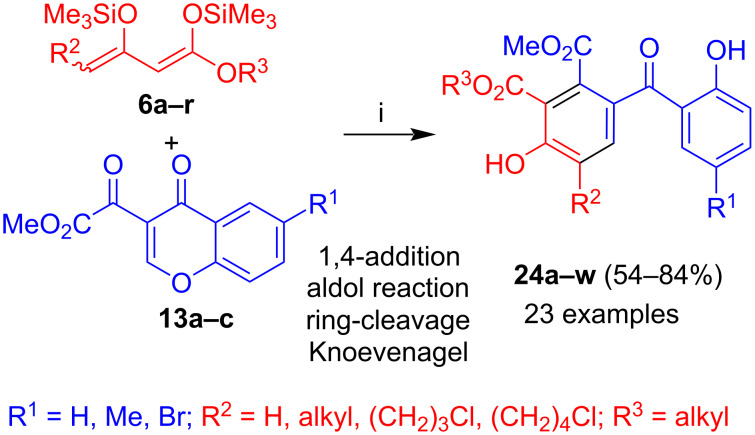
Synthesis of **24a–w**. Conditions: i, 1) **13a–c**, Me_3_SiOTf (0.3 equiv), 20 °C, 1 h; 2) **6a–r** (1.3 equiv), CH_2_Cl_2_, 0 → 20 °C, 12 h; 3) HCl (10%); 4) EtOH, *p*-TsOH (3 mol %), reflux, 5–10 h.

Reflux of an EtOH solution of **23a** in the presence of PTSA (*para*-toluenesulfonic acid) afforded benzophenone **24a** in 50% yield. The reaction of **6a–r** with **13a–c**, following the conditions discussed above for the synthesis of **23a–j**, and subsequent treatment with PTSA afforded benzophenones **24a–w** in a two-step on-pot procedure (Scheme 10) [39]. The formation of the products can be explained by 1,4-addition and subsequent aldol reaction to give products **24** and subsequent acid-catalyzed ring-cleavage and Knoevenagel reaction, similar to the formation of products **21** and **22**. The products were formed in 54–84% yields. Again, no systematic trend was observed for the yields. Good yields were observed for dienes containing various substituents located at carbon C-4 and for chromones containing a hydrogen, methyl or bromide at carbon C-6 of the chromone.

### 3-(Benzoyl)chromones

#### Dimethyl acetone-1,3-dicarboxylate

**Scheme 11:**
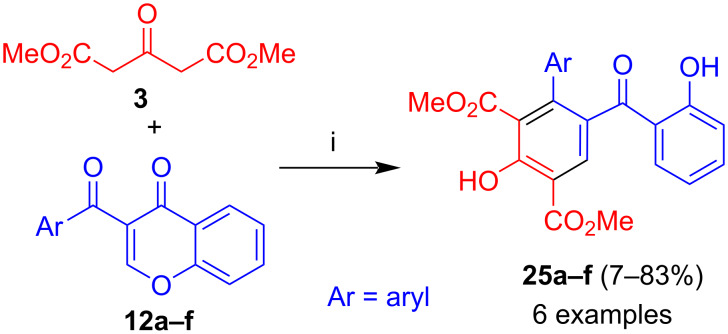
Synthesis of **25a–f**. Conditions: i, DBU (1.3 equiv), dioxane, 20 °C, 12 h.

The reaction of dimethyl acetone-1,3-dicarboxylate (**3**) with 3-aroylchromones **12a–f** afforded benzophenones **25a–f** (Scheme 11) [38]. A variety of aryl groups were tolerated, including 3- and 4-nitrophenyl and 3,5-dinitrophenyl. The formation of the product proceeds analogously to the reaction leading to benzophenones **19** and **21**. The yields were in most cases moderate to good. A poor yield (7%) was obtained from the chromone substituted by a 2-thienyl group which might be explained by the electron-rich nature of the thiophene and, thus, a reduced electrophilicity of the neighboring carbonyl group. Likewise, a poor yield (23%) was observed for a 2-fluorophenyl group, presumably due to steric hindrance because of the *ortho*-substituent. Excellent yields (70 and 83%) were obtained for a 3-nitro- and a 3,5-dinitrophenyl group, presumably due to the electron-withdrawing character of the nitro groups and an enhanced electrophilicity. A moderate yield (49%) was obtained for the phenyl derivative.

#### 1,3-Diphenylacetone

**Scheme 12:**
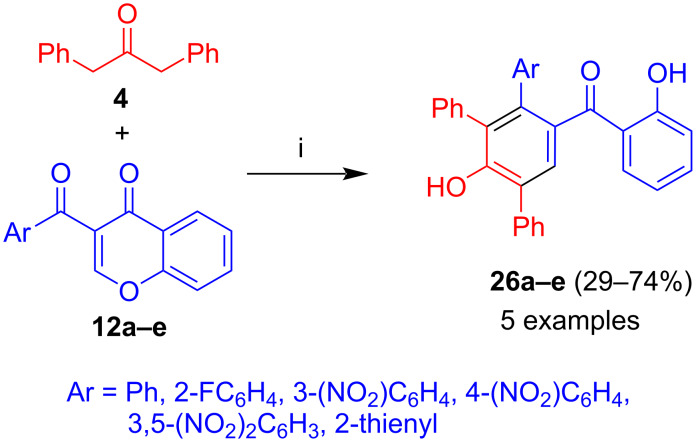
Synthesis of **26a–e**. Conditions: i, DBU (1.3 equiv), dioxane, 20 °C, 12 h.

Similarly, reaction of 1,3-diphenylacetone (**4**) with **12a–e** afforded benzophenones **26a–e** in moderate to good yields (Scheme 12) [38]. Similar to products **25**, a relatively low yield (29%) was observed for the chromone substituted by a 2-thienyl group. A low yield (32%) was also obtained for the phenyl derivative. Similar to products **25**, good yields (up to 74%) were obtained for products containing nitro groups.

### 3-(2-Nitrobenzoyl)chromones

#### Dimethyl acetone-1,3-dicarboxylate

**Scheme 13:**
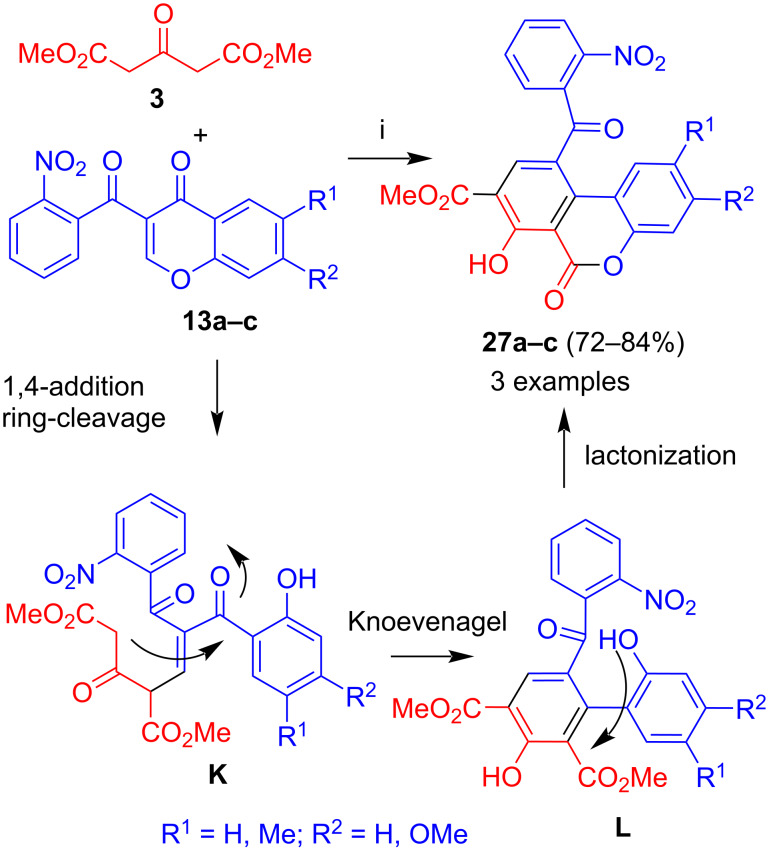
Synthesis of **27a–c**. Conditions: i, DBU (1.3 equiv), dioxane, 20 °C, 12 h.

A completely different result was obtained when chromones containing a 2-nitrobenzoyl group were employed. The reaction of dimethyl acetone-1,3-dicarboxylate (**3**) with 3-(2-nitrobenzoyl)chromones **13a–c** afforded benzocoumarines **27a–c** in good yields (Scheme 13) [38]. The formation of the products can be explained by 1,4-addition of **3** to the chromone and ring cleavage to give intermediate **K**. Subsequent Knoevenagel reaction by attack of the methylene carbon to the carbonyl group adjacent to the phenol moiety resulted in formation of intermediate **L** and lactonization gave the final products. The regioselectivity of the cyclization can be explained by steric hindrance of the carbonyl group adjacent to the 2-nitrophenyl moiety containing an *ortho*-substituent. Interestingly, in case of a 2-fluorophenyl group, the regioselectivity was different and product **26** was obtained (see Scheme 12). The yields were, in general, very good. The best yield (84%) was obtained for the reaction of 6-methyl-substituted 3-(2-nitrobenzoyl)chromone **13b**, presumably due to a better solubility of the product. However, the yields were in a similar range (72–84%).

#### 1,3-Diphenylacetone

**Scheme 14:**
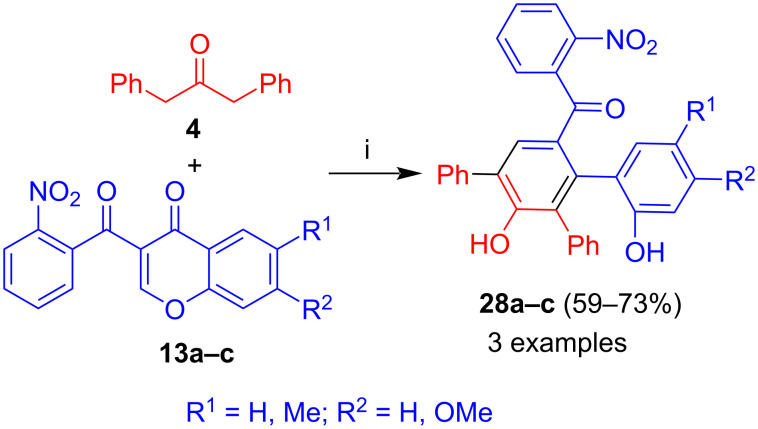
Synthesis of **28a–c**. Conditions: i, DBU (1.3 equiv), dioxane, 20 °C, 12 h.

The reaction of 1,3-diphenylacetone (**4**) with **13a–c** afforded biaryls **28a–c** (Scheme 14) [38]. Due to the absence of an ester group in starting material **4**, a lactonization in the final step was not possible. Similarly to products **27**, the best yield (73%) was obtained for the reaction of 6-methyl-substituted 3-(2-nitrobenzoyl)chromone **13b**. The yield of the product derived from unsubstituted 3-(2-nitrobenzoyl)chromone **13a** was slightly lower (59%).

### 3-(Perfluoroalkanoyl)chromones

#### Dimethyl acetone-1,3-dicarboxylate

**Scheme 15:**
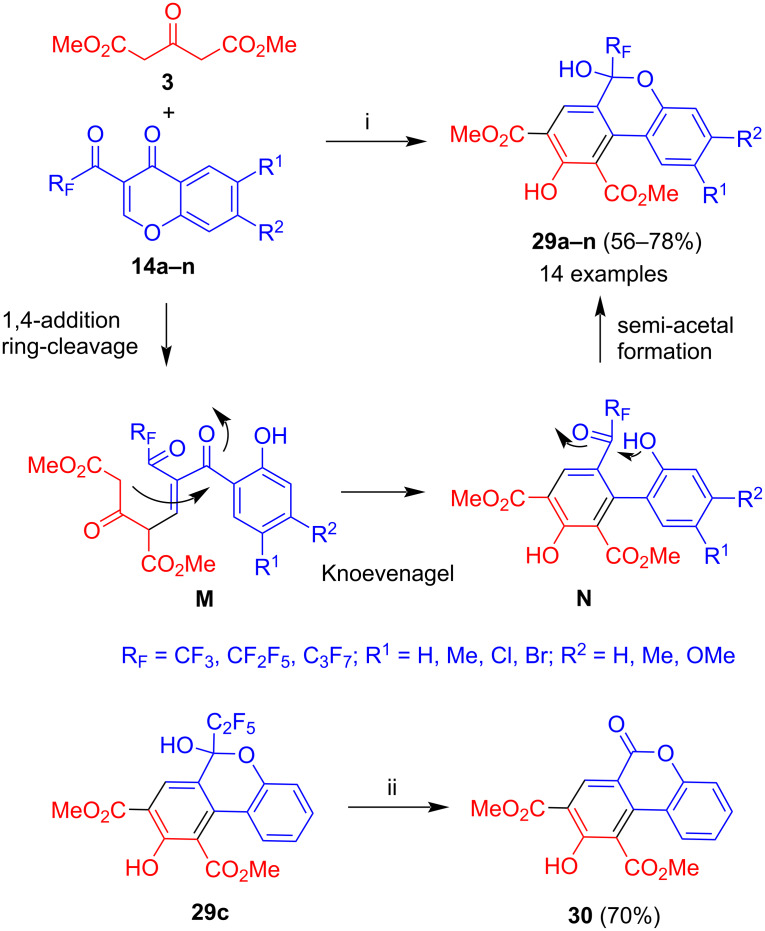
Synthesis of **29a–n** and **30**. Conditions: i, DBU (1.3 equiv), dioxane, 20 °C, 12 h; ii, 1) KOH, MeOH; 2) HCl (30%).

The reaction of dimethyl acetone-1,3-dicarboxylate (**3**) with 3-trifluoroacetyl- and 3-(perfluoroalkanoyl)chromones **14a–n** afforded hemiacetals **29a–n** in moderate to good yields (Scheme 15) [38]. The first step is again a 1,4-addition with subsequent ring cleavage of the chromone (intermediate **M**). Similar to the formation of products **27**, the cyclization proceeded by attack of the methylene carbon to the carbonyl group adjacent to the phenol moiety (intermediate **N**). This might be explained by the fact that the carbonyl adjacent to the perfluoroalkyl group exists as a hydrate which reduces significantly its electrophilicity. The products are finally generated by intramolecular formation of a semi-acetal. The yields were generally good (56–78%). The yields for pentafluoroethyl- and heptafluoropropyl-substituted derivatives were slightly better than the yields of trifluoromethyl-substituted derivatives which might be explained by a better solubility. For the other substituents of the chromone, no clear trend was observed. Product **29c** underwent cleavage of the perfluoroalkyl group upon treatment with potassium hydroxide in methanol to give benzocoumarine **30**.

#### 1,3-Bis(silyloxy)-1,3-butadienes

**Scheme 16:**
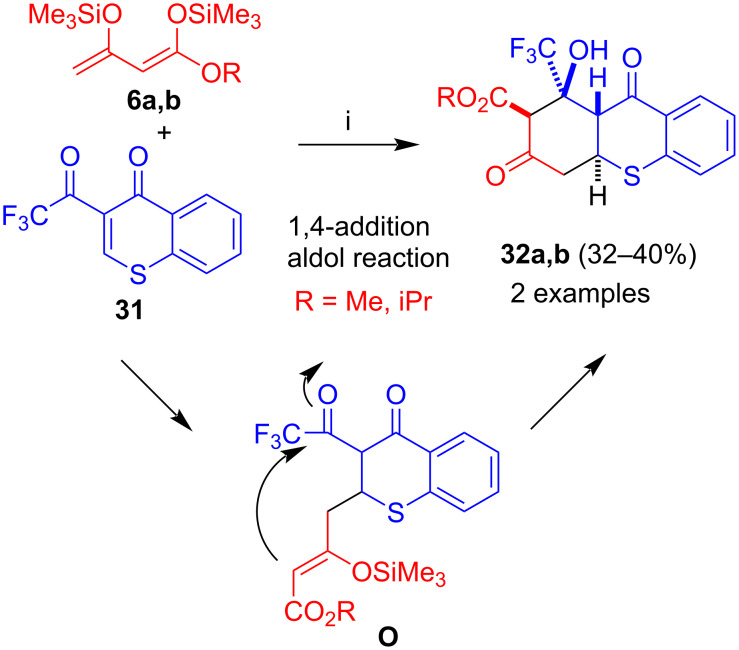
Synthesis of **32a**,**b**. Conditions: i, 1) **31**, Me_3_SiOTf (2.0 equiv), 20 °C, 1 h; 2) **6a**,**b** (3.0 equiv), CH_2_Cl_2_, 0 → 20 °C, 12 h; 3) HCl (10%).

The reaction of 3-(trifluoroacetyl)chromone (**14a**) with 1,3-bis(silyloxy)-1,3-butadienes proved not to be successful in our hands. However, reaction of 3-(trifluoroacetyl)thiochromone (**31**) with 1,3-bis(silyloxy)-1,3-butadienes **6a**,**b**, in the presence of Me_3_SiOTf, afforded products **32a**,**b** with excellent diastereoselectivity (Scheme 16) [40]. The formation of the products can be explained by 1,4-addition of the terminal carbon of the diene to the chromone to give intermediate **O** and subsequent attack of the central carbon atom of the 1,3-dicarbonyl unit (aldol reaction). No aromatization and extrusion of the hydroxy group was observed, because the electron-withdrawing character of the CF_3_ group which destabilizes the formation of a cation at the neighboring position. In addition, the carbon–sulfur bond was not cleaved, due to the lower leaving group ability of sulfur as compared to oxygen. The yields of the two products were moderate.

### 3-Halochromones

#### Dimethyl acetone-1,3-dicarboxylate

**Scheme 17:**
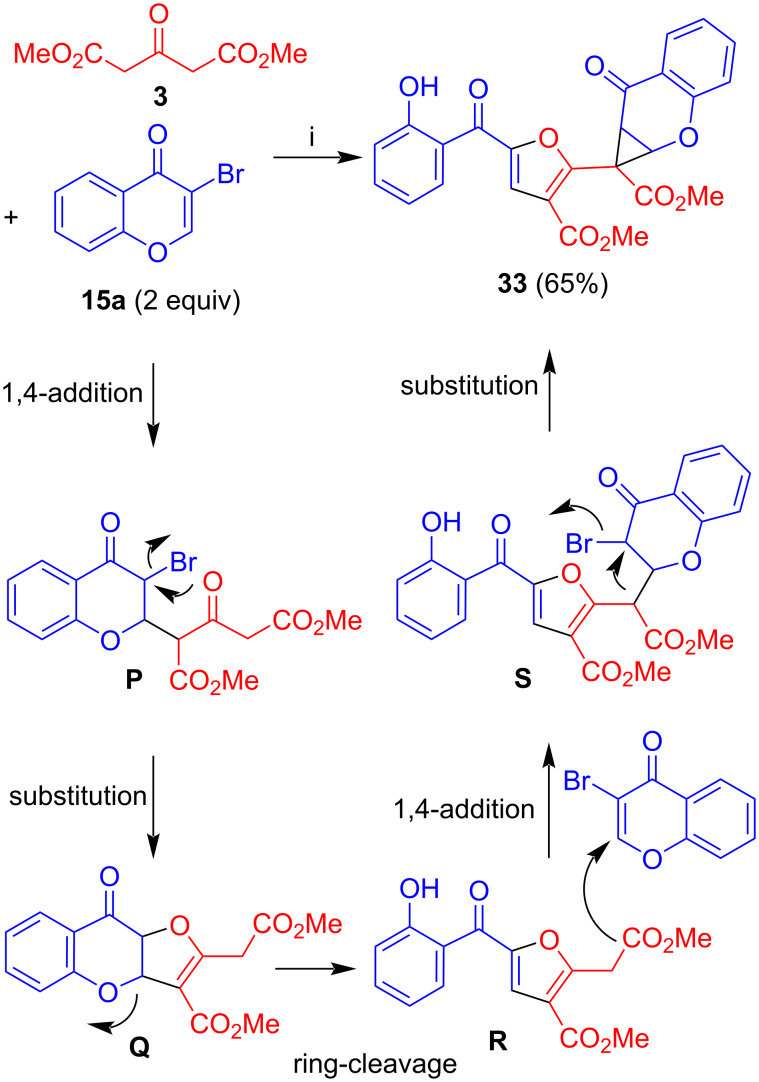
Synthesis of **33**. Conditions: i, DBU (1.3 equiv), THF, 20 °C, 12 h.

The reaction of dimethyl acetone-1,3-dicarboxylate (**3**) with 3-bromochromone (**15a**) afforded product **33** (Scheme 17) [32]. The first step is again a 1,4-addition to give intermediate **P**. Subsequent cyclization by intramolecular nucleophilic attack of the oxygen atom to the bromide afforded intermediate **Q** which underwent ring cleavage to give intermediate **R**. Subsequent 1,4-addition of the methylene carbon to a second molecule of **15a** gave intermediate **S** which underwent cyclization by attack of the methylene carbon to the bromide to give cyclopropane **33**.

#### 3-Ketoamides

**Scheme 18:**
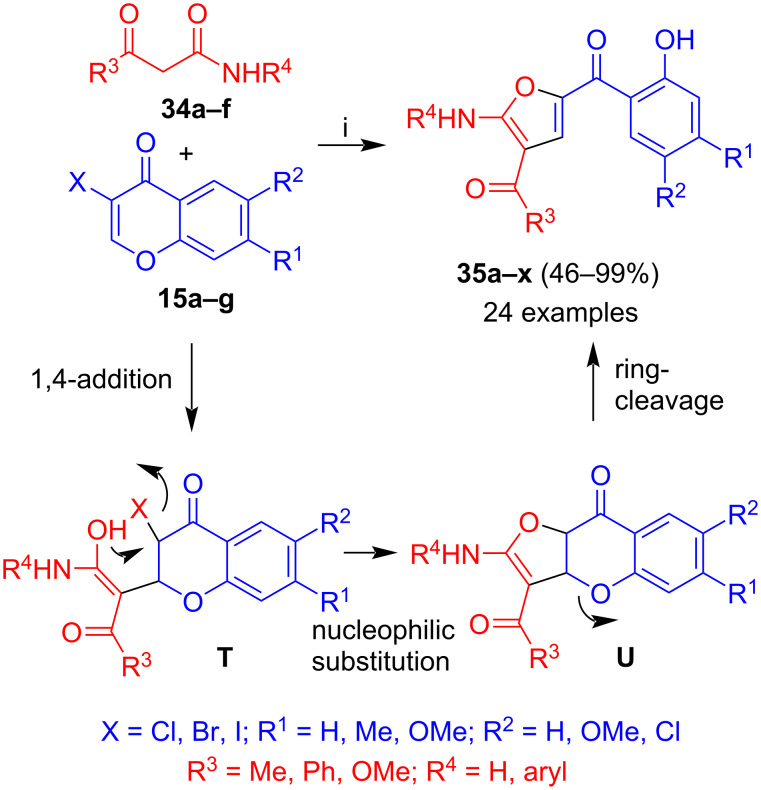
Synthesis of **35a–x**. Conditions: i, DBU (1.3 equiv), 1,4-dioxane, 20 °C, 12 h.

The reaction of 3-ketoamides **34a–f** with 3-halochromones **15a–g** afforded 2-(salicyloyl)furans **35a–x** in mostly good to excellent yields (Scheme 18) [41]. The reaction proceeds by 1,4-addition of the methylene carbon of **34** to the chromone to give intermediate **T**, cyclization by regioselective intramolecular nucleophilic attack of the oxygen to the halide to give intermediate **U** and subsequent ring-cleavage. The reaction of 3-ketoamide **34a** (R^3^ = Me, R^4^ = Ph) with 3-chloro-, 3-bromo-, and 3-iodochromone showed that the yields strongly depend on the type of halogen atom located at position 3 of the chromone. During the optimization of the conditions, the best yields were in many cases obtained when chloride was employed as the leaving group. However, for some other products the yields were slightly better in case of employment of 3-bromochromone. Most of the yields were higher than 70%, in several cases excellent (higher than 90%). Only in a few cases, the yields were moderate (46–53%). However, no clear trend was observed for the yields depending on the substituents. The reaction of 7-methoxy-substituted 3-halochromones were in most cases lower as compared to unsubstituted or chloro-substituted chromones, presumably due to the electron-donating character of the methoxy group and the lower electrophilicity of the chromone. But this trend was also not general. Similar yields were obtained for ketones (R^3^ = Me, Ph) or esters (R^3^ = OMe).

**Scheme 19:**
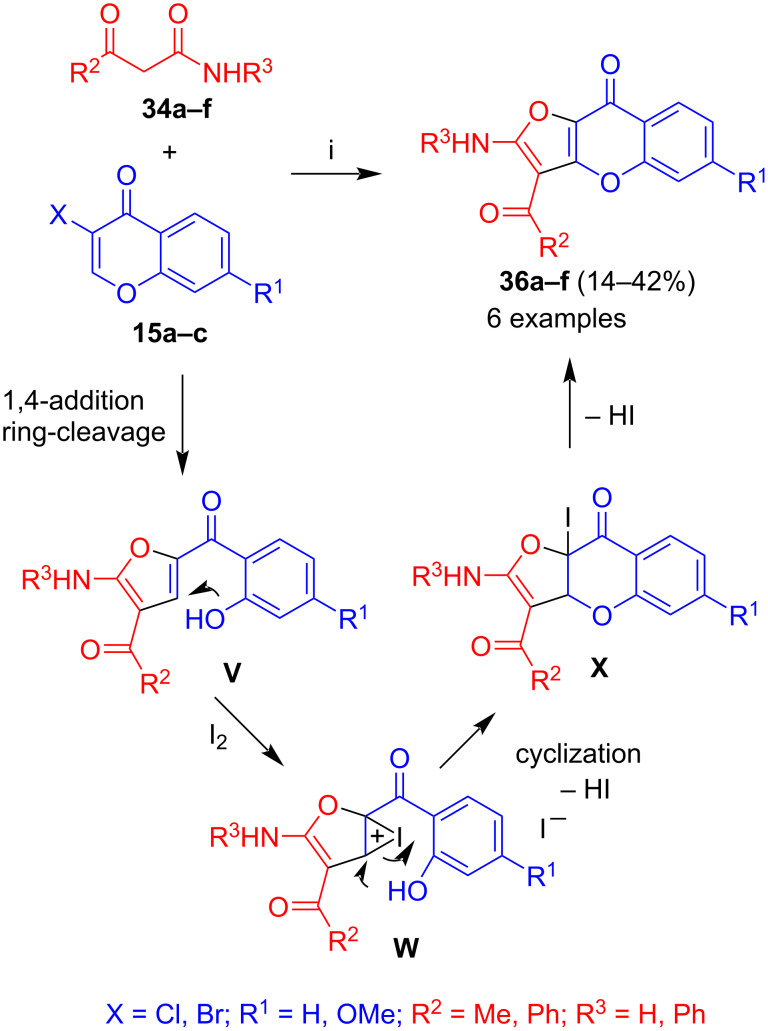
Synthesis of **36a–f**. Conditions: i, 1) DBU (1.3 equiv), 1,4-dioxane, 20 °C, 12 h; 2) I_2_ (2 equiv), DBU (3 equiv), MeCN or DMF.

Treatment of 2-(salicyloyl)furan **35a** with iodine in the presence of DBU afforded furo[3,2-*b*]chromen-9-one **36a** in 32% yield. Based on this observation, the two-step one-pot reaction of ketoamides **34a–f** with 3-halochromones **15a–c** was studied which afforded furo[3,2-*b*]chromen-9-ones **36a–f**, albeit, in only low to moderate yields (Scheme 19) [41]. The formation of these products can be explained by initial formation of the corresponding 2-(salicyloyl)furans **V**. Iodination of the latter afforded iodonium salt **W** which underwent cyclization by attack of the salicylate hydroxy group to give intermediate **X**. Extrusion of hydrogen iodide afforded the final product. Similarly to products **35**, relatively low yields were observed for reactions of methoxy-substituted 3-halochromones.

**Scheme 20:**
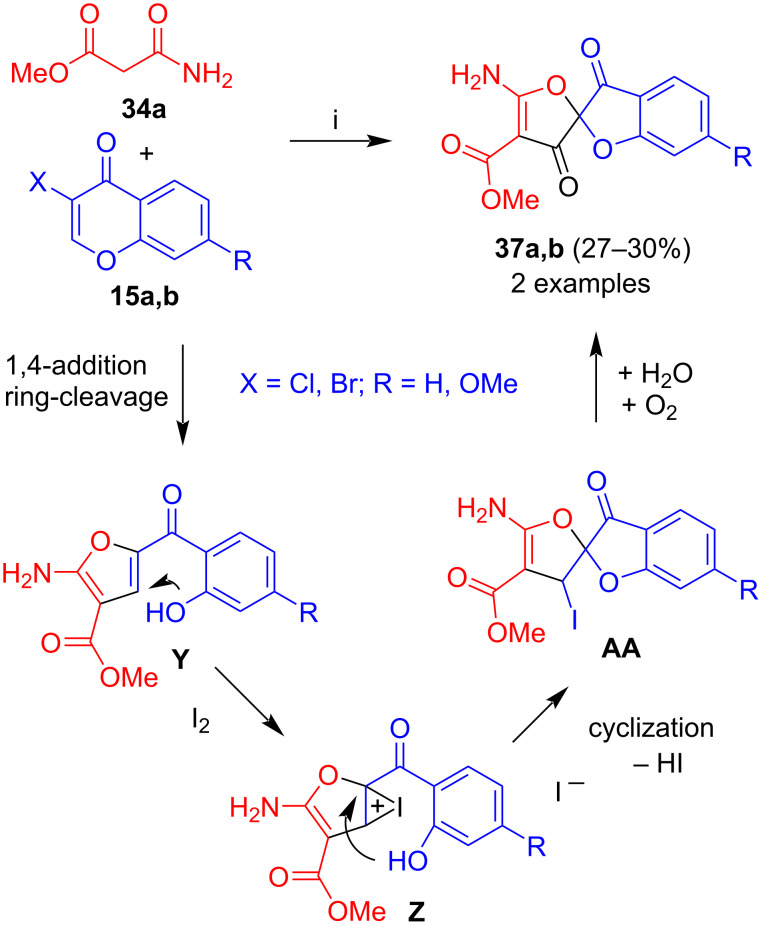
Synthesis of **37a**,**b**. Conditions: i, 1) DBU (1.3 equiv), 1,4-dioxane, 20 °C, 12 h; 2) I_2_ (2 equiv), DBU (3 equiv), MeCN or DMF. Then aqueous work-up at the air.

The two-step one-pot reaction of *N*-unsubstituted ketoamide **33a** with 3-halochromones **29a** and **31a** afforded spirocycles **36a**,**b**, albeit, in only moderate yields (Scheme 20) [41]. The formation of these products can be explained, similarly to the formation of **35a–f**, by generation of intermediate **Y** which afforded iodonium salt **Z** upon addition of iodine. Cyclization by attack of the salicylate hydroxy group to carbon C5 gave intermediate **AB**. Hydrolysis of the iodide upon aqueous work-up to give an alcohol and oxidation of the latter resulted in formation of the final product. The reason for the change of the regioselectivity of cyclization, as compared to the formation of products **36**, remains unclear at present.

**Scheme 21:**
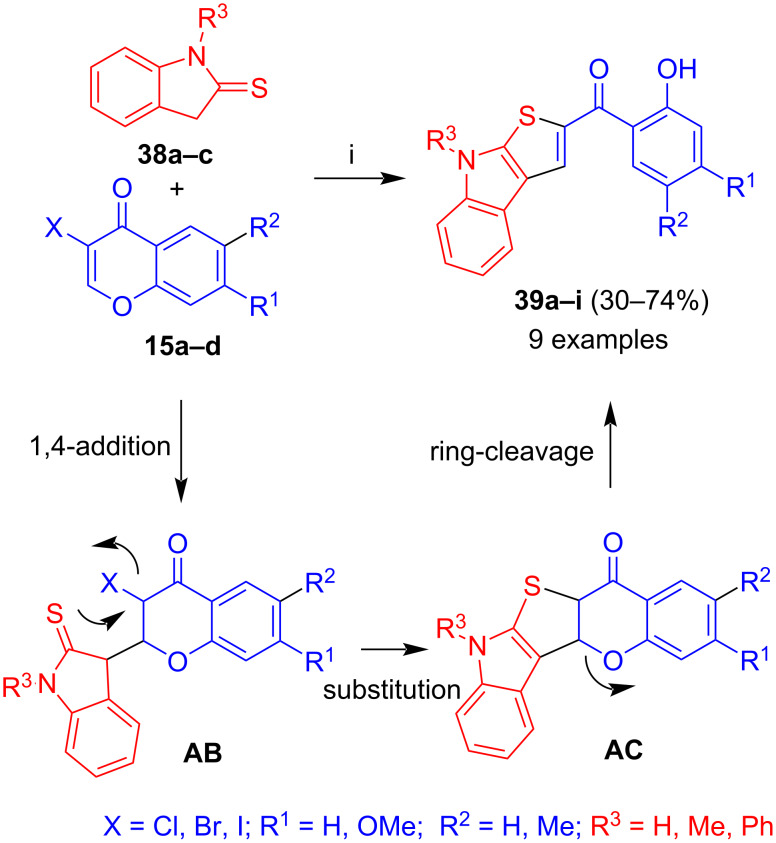
Synthesis of **39a–i**. Conditions: i, method A: DBU (1.3 equiv), 1,4-dioxane, 20 °C; method B: K_2_CO_3_ (4 equiv), DMF, 20 °C.

The reaction of 3-halochromones with 3*H*-indole-2-thiones (thiooxindoles) **38a–c** afforded 2-salicyloyl-8*H*-thieno[2,3-*b*]indoles **39a–i** in mostly moderate to good yields (Scheme 21) [41]. The formation of these products can be explained, similarly to the formation of products **35**, by 1,4-addition of the carbon atom of **38** to the chromone to give intermediate **AB**, cyclization by nucleophilic attack of the sulfur to the bromide (intermediate **AC**) and subsequent ring-cleavage. In most reactions, DBU was employed as the base. In case of products derived from *N*-unsubstititued 3*H*-indole-2-thiones (R^3^ = H), employment of potassium carbonate proved to be advantageous. Similarly to the formation of products **35**, the use of 3-chlorochromones gave the best yields. Likewise, the presence of a methoxy group located at the chromone moiety, resulted in a decrease of the yield, due to the electron-donating effect of the methoxy group.

### 3-Cyanochromones

#### Dimethyl acetone-1,3-dicarboxylate

**Scheme 22:**
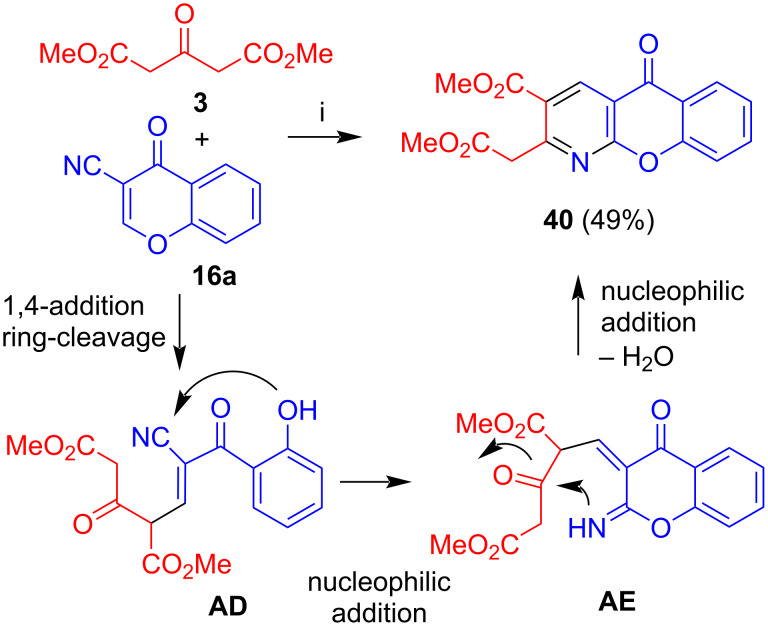
Synthesis of **40**. Conditions: i*,* piperidine, MeOH, CHCl_3_, reflux, 3 h.

The reaction of dimethyl acetone-1,3-dicarboxylate (**3**) with 3-cyanochromone (**16a**) afforded azaxanthone **40** in 49% yield (Scheme 22) [42–44]. The reaction again proceeds by 1,4-addition with subsequent ring cleavage of the chromone (intermediate **AD**). Subsequent attack of the methylene carbon to the nitrile gave intermediate **AE**. Attack of the imine to the carbonyl group and subsequent extrusion of water afforded product **40**. The reaction proved to be rather general and a variety of substituted 3-cyanochromones were successfully employed.

#### 1,3-Bis(silyloxy)-1,3-butadienes

**Scheme 23:**
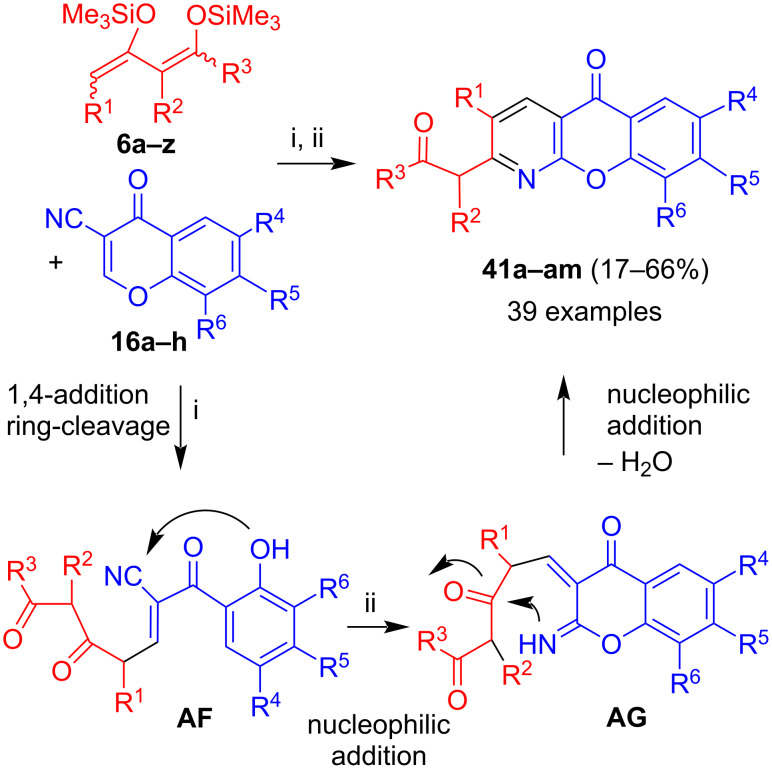
Synthesis of **41a–am**. Conditions: i*,* Me_3_SiOTf, CH_2_Cl_2_, 20 °C, 12 h, then: HCl (10%); ii*,* NEt_3_, EtOH or MeOH, 20 °C, 12 h, then: HCl (1 M).

The two-step reaction of 3-cyanochromones **16a–h** with 1,3-bis(silyloxy)-1,3-butadienes **6a–z** afforded azaxanthones **41a–am** (Scheme 23) [45–46]. The formation of the products can be explained by the mechanism discussed for the formation of azaxanthone **40** that involves intermediates **AF** and **AG**. For most products, the yields were in the range of 30 to 66%. Employment of dienes derived from 1,3-diketones (R^3^ = Me, Ph) proved to be unsuccessful, presumably due to the lower nucleophilicity of these dienes as compared to those derived from β-ketoesters (R^3^ = Oalkyl). Substituents located at carbon C-4 of the diene (R^1^) were generally tolerated as well. In fact, the best yield (66%) was obtained for the product derived from unsubstituted 3-cyanochromone (**16a**) and a diene containing a phenoxy group located at carbon C-4 (R^1^ = OPh). But in other cases, the yields were less. In fact, in some cases, cyano-substituted benzocoumarines, similar to products **18**, were formed as byproducts. Substituents located at carbon C-2 of the diene (R^2^) were tolerated, but the yields were lower, presumably due to the instability of these dienes [13]. In summary, no clear trend was observed for the influence of the substituents on the yields.

**Scheme 24:**
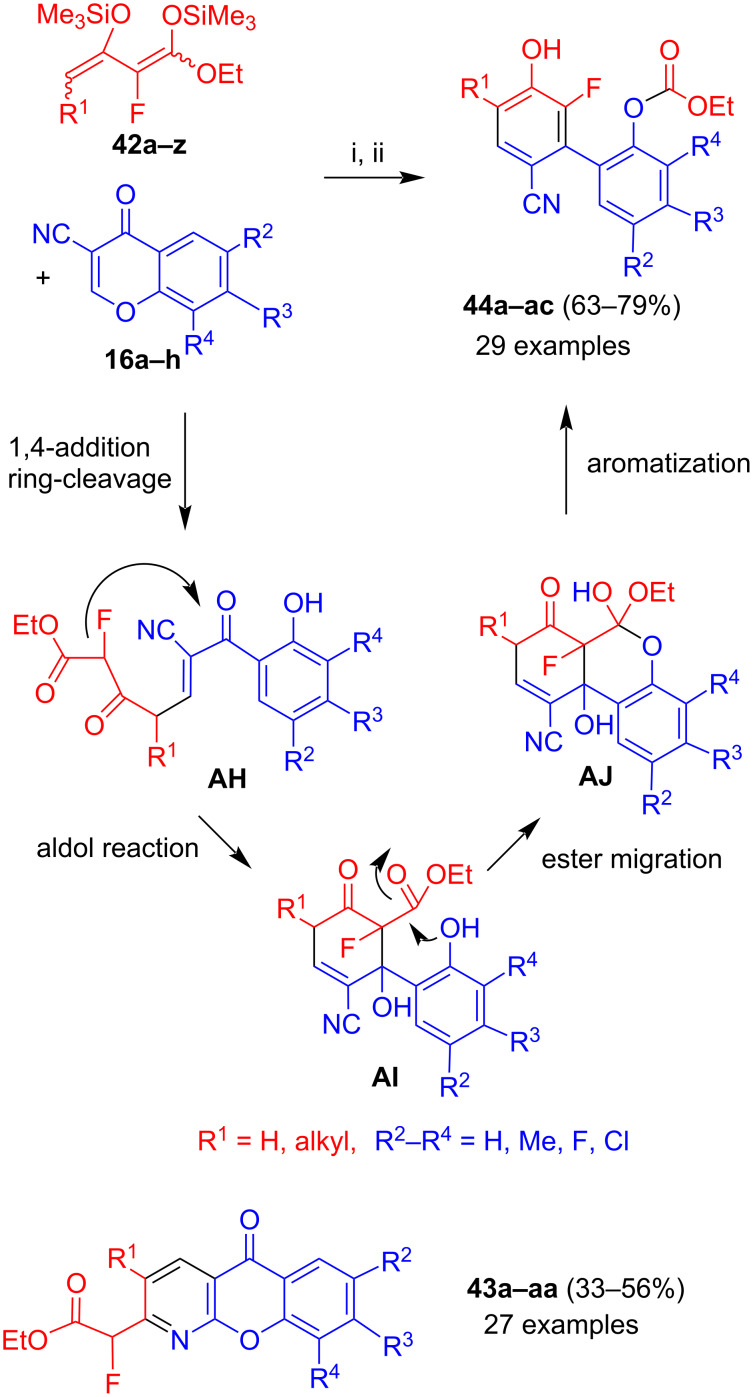
Synthesis of **43a–aa** and **44a–ac**. Conditions: i*,* Me_3_SiOTf, CH_2_Cl_2_, 20 °C, 12 h, then: HCl (10%); ii*,* NEt_3_, EtOH or MeOH, 20 °C, 12 h, then: HCl (1 M).

The reaction of 3-cyanochromones **16a–h** with 2-fluoro-1,3-bis(silyloxy)-1,3-butadienes **42a–z** afforded separable mixtures of azaxanthones **43a–aa** and biphenyls **44a–ac** (Scheme 24) [47]. In case of 4-unsubstituted 2-fluoro-1,3-bis(silyloxy)-1,3-butadienes, azaxanthones **43** were exclusively formed in 33–56% yields. A similar result was obtained for the corresponding 2-chloro-1,3-bis(silyloxy)-1,3-butadiene [48]. In case of 4-alkyl-2-fluoro-1,3-bis(silyloxy)-1,3-butadienes, biphenyls **44** were formed as the major products in 63–79% yields. In some cases, these products were exclusively formed. In other cases, azaxanthones **43** were isolated as minor products as well (7–20% yields). The formation of biphenyls **44** can be explained by 1,4-addition and ring-cleavage to give intermediate **AH**, aldol reaction (intermediate **AI**), migration of the ester group from the quaternary carbon to the phenolic oxygen atom (intermediate **AJ**) and aromatization. The steric influence of the fluorine atom and of the substituent R^1^ might have an influence on the conformation which facilitates the intramolecular aldol reaction at the expense of the nitrile addition. In the case of formation of biphenyls **44**, these products were generally obtained in good yields (63–79%). While the presence of a substituent located at carbon C-4 was mandatory, the exact type of substituent was not decisive for the yield. The substituents located at the chromone moiety also did not have a great impact on the yield.

## Conclusion

The present review highlighted reactions of chromones with 1,3-dicarbonyl compounds and 1,3-bis(silyloxy)-1,3-butadienes which can be regarded as electroneutral equivalents of dianions of 1,3-dicarbonyl compounds. The regioselectivity of these reactions depends mainly on the type of chromone. 2,3-Unsubstituted chromones undergo initial 1,4-addition and subsequent Knoevenagel condensation via the carbonyl group. In contrast, in case of 3-formylchromones the cyclization proceeds via the formyl group, due to its higher electrophilicity. The same regioselectivity was observed for 3-(methoxalyl)- and 3-benzoylchromones. In contrast, a cyclization via the carbonyl group of the chromone moiety was observed for 3-(2-nitrobenzoyl)chromone, presumably due to steric hindrance. The same selectivity was observed also for 3-trifluorochromone and 3-perfluoroalkanoylchromones which might be explained by the fact that the carbonyl adjacent to the perfluoroalkyl group exists as a hydrate which reduces significantly its electrophilicity. In case of 3-halochromones, the halide acts as a leaving group during the reaction. For 3-cyanochromones, a nucleophilic attack of chromone-derived hydroxy group to the nitrile was observed in most cases. However, a different reaction was observed for long chained 2-fluoro-1,3-bis(silyloxy)-1,3-butadienes. In conclusion, the interaction of activated carbonyl compounds with chromones offer a great variety of different reaction pathways leading to various types of products with potential applications in materials science and medicinal chemistry.

The domino reactions presented, which involve mostly the combination of 1,4-additions of the carbonyl compounds to the chromone moiety with ring cleavage reactions, allow for the synthesis of products which are not readily available by other methods. The benefit for the employment of chromones as building blocks is their dense functionalization in combination with their (commercial) availability. On the other hand, highly substituted or functionalized chromones have to be prepared in multi-step syntheses. In general, the substitution pattern of the chromone does not seem to have a great influence on the yields which is beneficial for studies related to the scope and the synthesis of compound libraries. The use of dimethyl acetone-1,3-dicarboxylate as a starting material has the advantage that it is commercially available, stable and easy to handle. The use of 1,3-bis(silyloxy)-1,3-butadienes is, on the other hand, significantly more difficult and usually requires the use of the Schlenck technique to avoid hydrolysis of the moisture sensitive compounds. The handling of these reagents, which have to be stored at low temperature, requires some experience. In fact, the synthesis of the dienes has to proceed with full conversion and in high yield as the they cannot be purified by chromatography. Distillation is also problematic, as dienes containing substituents located at carbon atom C-2 readily undergo isomerization reactions. The reactions of 1,3-bis(silyloxy)-1,3-butadienes with chromones requires the action of trimethylsilyl trifluoromethanesulfonate (TMSOTf) which is a relatively expensive reagent.

In future studies, especially the chemistry of 3-methoxalyl- and 3-nitrochromones should be further explored. In case of the methoxalyl derivatives, so far not many synthetic applications have been developed. In the future, it will be interesting also to study reactions of 3-nitrochromone with carbonyl compounds. In this context, biological properties of the products will be interesting, as the nitro group can be transformed to the biologically important amino group.

## Data Availability

Data sharing is not applicable as no new data was generated or analyzed in this study.

## References

[1] Tietze L F, Beifuss U (1993). Angew Chem, Int Ed Engl.

[2] Padwa A, Bur S K (2007). Tetrahedron.

[3] Pellissier H (2006). Tetrahedron.

[4] Nicolaou K C, Edmonds D J, Bulger P G (2006). Angew Chem, Int Ed.

[5] Tietze L F (1996). Chem Rev.

[6] Langer P, Döring M (2002). Eur J Org Chem.

[7] Langer P, Freiberg W (2004). Chem Rev.

[8] Bellur E, Feist H, Langer P (2007). Tetrahedron.

[9] Dang T T, Dang T T, Langer P (2011). Synlett.

[10] Langer P (2023). Synlett.

[11] Langer P (2023). Synlett.

[12] Langer P (2023). Synlett.

[13] Langer P (2002). Synthesis.

[14] Langer P (2006). Synlett.

[15] Feist H, Langer P (2007). Synthesis.

[16] Langer P (2007). Eur J Org Chem.

[17] Langer P (2009). Synlett.

[18] Nawaz M, Sher M, Langer P (2010). Synlett.

[19] Karapetyan G, Dang T T, Sher M, Ghochikyan T V, Saghyan A, Langer P (2012). Curr Org Chem.

[20] Dang T T, Kelzhanova N K, Abilov Z A, Turmukhanova M Z, Langer P (2012). Synlett.

[21] Hassan Z, Langer P (2019). Synlett.

[22] Langer P (2021). Synlett.

[23] Feist H, Langer P (2008). Synthesis.

[24] Madhav H, Jameel E, Rehan M, Hoda N (2022). RSC Med Chem.

[25] Fu L, Wan J-P (2019). Asian J Org Chem.

[26] Keri R S, Budagumpi S, Pai R K, Balakrishna R G (2014). Eur J Med Chem.

[27] Gaspar A, Matos M J, Garrido J, Uriarte E, Borges F (2014). Chem Rev.

[28] Sharma S K, Kumar S, Chand K, Kathuria A, Gupta A, Jain R (2011). Curr Med Chem.

[29] Langer P (2007). Synlett.

[30] Langer P (2022). Synlett.

[31] Langer P (2022). Synlett.

[32] Terzidis M A, Tsoleridis C A, Stephanidou-Stephanatou J, Terzis A, Raptopoulou C P, Psycharis V (2008). Tetrahedron.

[33] Langer P, Saleh N N R, Freifeld I (2002). Chem Commun.

[34] Appel B, Saleh N N R, Langer P (2006). Chem – Eur J.

[35] Appel B, Rotzoll S, Kranich R, Reinke H, Langer P (2006). Eur J Org Chem.

[36] Wolf V, Adeel M, Reim S, Villinger A, Fischer C, Langer P (2009). Eur J Org Chem.

[37] Holtz E, Albrecht U, Langer P (2007). Tetrahedron.

[38] Iaroshenko V O, Savych I, Villinger A, Sosnovskikh V Y, Langer P (2012). Org Biomol Chem.

[39] Iaroshenko V O, Bunescu A, Spannenberg A, Supe L, Milyutina M, Langer P (2011). Org Biomol Chem.

[40] Iaroshenko V O, Bunescu A, Sevenard D V, Sosnovskikh V Y, Langer P (2015). Russ Chem Bull.

[41] Savych I, Gläsel T, Villinger A, Sosnovskikh V Y, Iaroshenko V O, Langer P (2015). Org Biomol Chem.

[42] Ghosh C, SinhaRoy D K, Mukhopadhyay K K (1979). J Chem Soc, Perkin Trans 1.

[43] Kubo K, Ukawa K, Kuzuna S, Nohara A (1986). Chem Pharm Bull.

[44] Zheng H, Lin G, Weng L L (1998). Indian J Chem.

[45] Langer P, Appel B (2003). Tetrahedron Lett.

[46] Rashid M A, Rasool N, Appel B, Adeel M, Karapetyan V, Mkrtchyan S, Reinke H, Fischer C, Langer P (2008). Tetrahedron.

[47] Ibad M F, Abid O-u-R, Adeel M, Nawaz M, Wolf V, Villinger A, Langer P (2010). J Org Chem.

[48] Reim S, Adeel M, Hussain I, Yawer M A, Ahmed Z, Villinger A, Langer P (2008). Tetrahedron Lett.

